# Remote Monitoring of Sympathovagal Imbalance During Sleep and Its Implications in Cardiovascular Risk Assessment: A Systematic Review

**DOI:** 10.3390/bioengineering11101045

**Published:** 2024-10-19

**Authors:** Valerie A. A. van Es, Ignace L. J. de Lathauwer, Hareld M. C. Kemps, Giacomo Handjaras, Monica Betta

**Affiliations:** 1 MoMiLab Research Unit, IMT School for Advanced Studies Lucca, 55100 Lucca, Italy; giacomo.handjaras@imtlucca.it (G.H.); monica.betta@imtlucca.it (M.B.); 2 Department of Cardiology, Máxima Medical Centre, 5504 DB Veldhoven, The Netherlands; 3 Department of Industrial Design, Eindhoven University of Technology, 5600 MB Eindhoven, The Netherlands

**Keywords:** remotepatient monitoring, nocturnal sympathetic overdrive, sleep, cardiovascular risk, autonomic nervous system, cardiovascular health technologies

## Abstract

Nocturnal sympathetic overdrive is an early indicator of cardiovascular (CV) disease, emphasizing the importance of reliable remote patient monitoring (RPM) for autonomic function during sleep. To be effective, RPM systems must be accurate, non-intrusive, and cost-effective. This review evaluates non-invasive technologies, metrics, and algorithms for tracking nocturnal autonomic nervous system (ANS) activity, assessing their CV relevance and feasibility for integration into RPM systems. A systematic search identified 18 relevant studies from an initial pool of 169 publications, with data extracted on study design, population characteristics, technology types, and CV implications. Modalities reviewed include electrodes (e.g., electroencephalography (EEG), electrocardiography (ECG), polysomnography (PSG)), optical sensors (e.g., photoplethysmography (PPG), peripheral arterial tone (PAT)), ballistocardiography (BCG), cameras, radars, and accelerometers. Heart rate variability (HRV) and blood pressure (BP) emerged as the most promising metrics for RPM, offering a comprehensive view of ANS function and vascular health during sleep. While electrodes provide precise HRV data, they remain intrusive, whereas optical sensors such as PPG demonstrate potential for multimodal monitoring, including HRV, SpO2, and estimates of arterial stiffness and BP. Non-intrusive methods like BCG and cameras are promising for heart and respiratory rate estimation, but less suitable for continuous HRV monitoring. In conclusion, HRV and BP are the most viable metrics for RPM, with PPG-based systems offering significant promise for non-intrusive, continuous monitoring of multiple modalities. Further research is needed to enhance accuracy, feasibility, and validation against direct measures of autonomic function, such as microneurography.

## 1. Introduction

The autonomic nervous system (ANS) primarily oversees the homeostatic balance of the body through control centers located in the hypothalamus, brainstem, the spinal cord, and peripheral ganglia [1,2]. Via reflexes, the ANS receives sensory inputs mainly from thoracic and abdominal viscera and exerts its effects on nearly every tissue across the body, fine-tuning all the involuntary physiological processes [3,4]. The ANS operates through the complementary activity of its main branches, namely the sympathetic and the parasympathetic nervous systems. In general, the sympathetic system activates organs or tissues to facilitate strenuous physical activity, while the parasympathetic system predominates during periods of rest [3]. Concerning the role of ANS in regulating the cardiovascular (CV) functions, the sympathetic system elevates heart rate (HR) and blood pressure (BP) by increasing the force, and the rate of contraction (i.e., inotropic and chronotropic effects, respectively) as well as by enhancing conduction (i.e., dromotropic effect) of the heart, and synergistically reducing the diameter of arterioles at a peripheral level. On the other hand, the parasympathetic system action decreases HR and, unlike sympathetic activity, has minimal effect on myocardial contractility and on the size of small blood vessels [5].

In a physiological state, the sympathetic and parasympathetic branches work together to maintain internal homeostasis according to a dynamic equilibrium between the two [6]. The disruption in this subtle dynamic equilibrium is termed a sympathovagal imbalance and often arises in the form of an increased sympathetic tone and vagal withdrawal [7,8,9]. Importantly, a state of sympathetic overactivity seems to promote mechanical, inflammatory, and hemodynamic alterations in the heart and in the vascular system [10,11,12,13]. Therefore, a prolonged sympathetic overdrive has been recognized as a factor implicated in the pathogenesis, progression and prognosis of different CV and metabolic diseases including hypertension, myocardial infarction, cardiac arrhythmias, congestive heart failure, obesity, and diabetes [14,15,16,17,18,19,20,21,22,23,24,25,26]. The determinants potentially contributing to this sympathetic dysregulation are multiple and encompass alterations in autonomic reflex pathways [27], central autonomic neuroanatomical sites [28] and hormonal control processes [29].

Despite its myriad implications, the early causes that induce a prolonged state of sympathoexcitation and parasympathetic withdrawal remain elusive. Considering the clinical implications of a sympathovagal imbalance [30], there is an urgency to develop methods capable of monitoring and detecting prodromal autonomic alterations in real life. An important principle that has been advocated is to focus on nocturnal autonomic activity, leveraging the absence of many confounders to physiological autonomic activity (e.g., daily living and work activities), and the ANS’s dominance over the central nervous system during sleep [2,31,32].

During the night, ANS activity undergoes significant changes in association with sleep stage transitions [33,34,35], as well as in correspondence to both physiological and pathological sleep-related events able to trigger abrupt and transient sympathetic activations (e.g., cortical arousal, apnea/hypopnea events, limb-movement) [36,37,38]. Specifically, sleep-related disorders (i.e., insomnia, sleep breathing disorders or periodic leg movement syndrome) can induce a nocturnal autonomic over-stimulation [2,39,40], potentially resulting in sustained modifications of daytime sympathetic nervous activity [17,41,42,43]. Importantly, these sleep-related pathologies, characterized by a heightened sympathetic dominance, are typically associated with cerebrovascular, CV, and metabolic disorders [12,39,44,45,46,47]. This underscores the importance of identifying early states of nocturnal sympathetic overdrive, during which mechanical and inflammatory changes typically observed in CV diseases are not yet permanent and may be reversible with effective non-pharmacological and pharmacological intervention strategies [30]. Further exploration in this direction should commence with non-invasive, broad-spectrum assessments of sympathetic versus parasympathetic activation levels. However, mapping nocturnal ANS function efficiently poses theoretical and methodological challenges. Consequently, there is an urgent need for technologies, including both hardware and algorithms, that enable continuous remote patient monitoring (RPM) of sympathetic overactivity during sleep. These technologies must ensure data accuracy, minimize sleep disruption, be cost-effective for widespread use, and allow for continuous data collection. By collecting continuous data, RPM can identify patterns in ANS activity, providing a more reliable assessment of changes over time [48]. This capability allows for the early detection of ANS dysregulation, which is linked to early markers of CV pathologies, thereby possibly facilitating timely interventions [49,50].

The aim of this systematic review was threefold. First, we examined the literature to categorize and describe current methods for monitoring ANS activity during sleep. Secondly, we highlighted how alterations of nocturnal autonomic functions are linked to CV implications in the context of clinically relevant monitoring systems. Finally, we evaluated and compared the identified methods for their feasibility based on metric compatibility, intrusiveness, data accuracy, continuity, and practical considerations.

## 2. Materials and Methods

Guidelines according to the Preferred Reporting Items for Systematic Reviews and Meta-Analyses (PRISMA) were followed [51].

### 2.1. Systematic Literature Search and Article Selection

**Figure 1:**
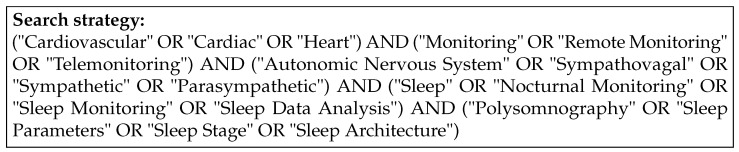
Search strategy to identify related publications following the PICOS specifications.

To identify the related publications the Population, Intervention, Comparator, Outcome (PICO) framework was applied while searching through the titles and abstracts in PubMed, SpringerLink, ACM Digital Library, Scopus, and IEEE Xplore, accessed on 21 December 2023, as shown in Figure 1 [52].

The search strategy was designed to minimize selection bias and ensure comprehensive coverage of relevant studies. We followed the PICO framework, applying specific inclusion criteria to ensure that the reviewed studies were directly relevant to our research objectives. Studies were included based on the following criteria:

- Population (P): Research focused on measuring nocturnal ANS activity with direct relevance to CV implications.
- Intervention (I): Studies utilizing novel or existing modalities, metrics, or algorithms capable of being integrated into RPM systems.
- Comparison (C): Traditional RPM methods (e.g., spot measurements of weight, BP, HR, or symptoms checklists) used as benchmarks.
- Outcome (O): Evaluation of new algorithms and technologies for identifying and monitoring nocturnal sympathetic overdrive and its CV implications.

To ensure a robust and unbiased selection of studies for our review, two independent reviewers (V.A.A.v.E. and I.L.J.d.L.) conducted a dual screening of all titles and abstracts. Each reviewer independently assessed the studies, documenting their findings in an Excel sheet, which included key information on the studies’ relevance based on the predefined inclusion criteria. This method allowed the reviewers to maintain objectivity and transparency throughout the process, minimizing potential bias during the selection of studies for full-text review.

The criteria for study inclusion were explicitly defined to align with the study’s objectives, focusing on non-invasive monitoring of the ANS, CV implications, and sleep-related factors. Studies were excluded if they did not collect vital signs during sleep, used invasive sensors, did not adequately consider the physiology of the ANS in relation to CV implications, were not retrievable or accessible, or had limited clinical relevance due to small sample sizes (e.g., n = 1). This systematic approach ensured that the studies included in the review were relevant and met the specific aims of the research.

### 2.2. Data Extraction, Risk of Bias Assessment Tool and Quality Scales

Both reviewers extracted data using an electronic spreadsheet containing the following categories:

- Brief description of the study design, population, and experimental setup.
- Vital signs measured including the metrics related to autonomic regulation.
- Technical details of the technology used including its modality, metrics, device location during measuring, and the application in the related study.
- Consideration of ANS physiology related to CV implication.
- Primary and secondary outcomes.
- Feasibility assessment for RPM integration.

In instances of disagreements during the extraction process, the two reviewers (V.A.A.v.E. and I.L.J.d.L.) engaged in discussions until consensus was reached. If necessary, a third reviewer was consulted. Regular quality checks were implemented to ensure consistency between reviewers. Our methodology was crafted to uphold the integrity of the review process, aiming to provide a comprehensive and unbiased assessment of technologies with the potential for RPM of nocturnal autonomic regulation in an unobtrusive manner. This approach is geared toward delivering clinically relevant information for CV risk assessment, patient monitoring, and early interventions.

The evaluation of bias in studies is essential, as it can elucidate variations in the outcomes of studies incorporated into a systematic review. The risk of bias was assessed independently by both reviewers using Cochrane’s recommended tool for evaluating bias and applicability in primary diagnostic accuracy studies within systematic reviews—the QUADAS-2 tool [53]. Employing a domain-based assessment approach, evaluations were conducted separately for four domains, namely: ‘Patient Selection’ (D1), ‘Index Test’ (D2), ‘Reference Standard’ (D3), and ‘Flow and Timing’ (D4). Ratings such as ‘low risk’, ‘high risk’, ‘some concerns’, or ‘no information’ were assigned to each domain based on these appraisals. The overall risk of bias was determined by aggregating the assessments across the four domains.

## 3. Results

### 3.1. Study Selection

**Figure 2:**
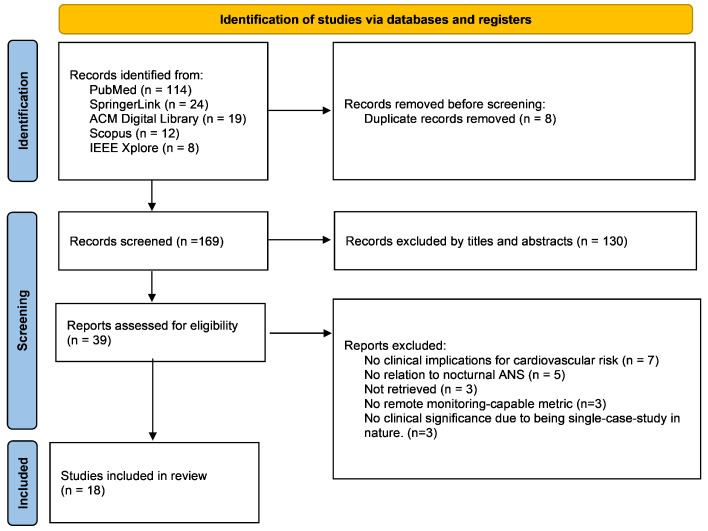
The PRISMA [51] four-phase flow diagram delineating the procedure for the identification and selection of studies included in the qualitative synthesis.

Figure 2 depicts the PRISMA four-phase flow diagram illustrating the sequential stages involved in identifying and selecting the studies. A total of 177 abstracts were retrieved from five databases. Following a manual search for duplicate records, eight instances were identified and subsequently removed. After an initial screening of 169 articles based on their titles and abstracts, 130 records were excluded, with details on titles, DOIs, and reasons for exclusion provided in Table S1 in the Supplementary Materials. Upon conducting a comprehensive analysis of the entire text of the 39 studies, 21 were excluded for various reasons, such as not being retrieved, lacking clinical pertinent CV implications (e.g., studies which were developing remote techniques for detecting falling asleep and awakening time, not focusing on the CV track), having no direct link to nocturnal ANS activity (e.g., studies which created algorithms based on wakefulness measurements), presenting metrics incompatible for integration into a RPM system (e.g., studies that were using high-density EEG), and lacking clinical relevance due to being single-case-study in nature. In the end, we included 18 articles for full-text screening.

A summary of the study characteristics, as well as an overview of all the technologies, their applications and the associated CV implications, are presented in Table 1. Importantly, besides the included cross-sectional and validation studies, there was one prospective cohort study by Costa et al. (2021) [54], and three reviews by Matar et al. (2018) [55], Murali et al. (2003) [56], and Park and Choi (2019) [57].

### 3.2. Technologies and Metrics for Non-Invasive Monitoring of Nocturnal Autonomic Nervous System Activity

The included studies adopted diverse modalities and metrics for assessing nocturnal autonomic dysfunction. These ranged from more intrusive methods using electrodes/sensors in direct contact with the body including electrocardiogram (ECG), photoplethysmography (PPG) and peripheral arterial tone (PAT), to less obtrusive options such as smartwatches. Additionally, completely unobtrusive sensors, like ballistocardiography (BCG) load cells affixed to the bed legs, optical fibers seamlessly integrated into the textile of bed sheets or clothes, as well as complete contactless radars and cameras, were described.

#### 3.2.1. Intrusive Modalities

#### Electrodes

Depending on the placement of electrodes on various body parts, different modalities can be generated, such as electroencephalography (EEG), ECG, electrodermal activity (EDA), electromyography (EMG), and electrooculography (EOG). These modalities provide metrics related to neural activity, heart electrical activity, skin conductance, muscle electrical activity, and eye movements, respectively. Polysomnography (PSG), which uses many of the above mentioned techniques, is considered the gold standard for clinical sleep monitoring, aiding in defining sleep stages and diagnosing disorders like sleep-related breathing disorders [58]. While PSG may provide insights on autonomic dysfunction activity in sleep disorders, it requires significant time, hospitalization, and qualified staff, making it costly and less accessible. This limits its adoption in the clinical population leading to a low detection rate of autonomic dysfunction during sleep, with undiagnosed cases of moderate to severe sleep-related breathing disorders [59]. Therefore, there is a need for a simple, fast, and inexpensive test for screening autonomic dysregulation during the night.

**Table 1:** Overview and summary of technologies compatible with remote monitoring systems from the included publications.

Study	Design and Population	Modality	Metrics	Device Location	Application	Cardiovascular Implications
Baek and Cho, 2019 [60]	Experimental, n = 16 healthy sleep, n = 15 stress speech task, n = 5 free living 24 h	PPG	HRV index derived from oscillation equation-based frequency algorithm	Wrist	Continuous HRV monitoring, overcoming motion artifacts	Monitoring risk of CV disease based on imaging continuous ANS dynamics in daily life
Cabiddu et al., 2015 [61]	Observational cross-sectional, n = 18 obese, n = 20 healthy	Electrodes (ECG)	HRV: SE, LZC, DFA	Chest	Imaging of adaptive capabilities and ANS stability	Obesity associated with decreased HRV complexity and sympathovagal imbalance during NREM sleep, posing CV risk
Carek and Holz, 2018 [62]	Experimental, n = 5 healthy sleep for 4 nights	Unobstructive BCG and PPG	PTT-based BP	Legs	Continuous non-invasive monitoring of BP	Holistic assessment of hypertension requires 24 h BP since patients might exhibit nocturnal hypertension without signs during the day
Costa et al., 2021 [54]	Prospective cohort, n = 1858	Electrodes (ECG)	HRF: PIP, ALS, PNNLS, PNNSS	Chest	Imaging of abnormal sinoatrial dynamics	HRF better predicts AF than standard HRV parameters, varies with sleep stages and sympathetic/parasympathetic activities
Jung et al., 2016 [63]	Validation, n = 20 non-nocturnal hypoxemia, n = 76 nocturnal hypoxemia	Unobstructive BCG	HRV: SDNN, RMSSD, NN50, pNN50, LF, HF, LF/HF, SD1, SD2, SD1/SD2	Beneath bed’s legs (load cell); under mattress at dorsal surface (PVDF- or EMFi film sensor)	Imaging of nocturnal cardiac sympathetic activation	LF component of HRV highly predicts ODI, reflecting sympathetic modulation of HR
Lee et al., 2020 [64]	Validation, n = 165 OSA, n = 59 healthy	Electrodes (EEG, ECG), PPG finger cuff	MLP neural network trained on multiple features	Head (EEG), chest (ECG), finger (PPG)	Detection of sleep-disordered breathing with sympathetic overdrive	MLP neural networks classify sleep-disordered breathing posing CV risk, based on SpO2mean, and SpO2min
Matar et al., 2018 [55]	Review	IR- and RGB camera, unobstructive BCG, radar, optical fibers, EEG, PPG	HR, HRV, RRV, actigraphy, EDA	Sensor in pillow (EDA), contactless at bedside (camera, radar), wrist (EDA, PPG), head (EEG)	Sleep staging, quality check, and OSA detection	Sleep stage changes linked to neural circulatory control, hemodynamics measured by HRV, respiratory rate
Mayer et al., 2019 [65]	Validation, n = 24 suspected OSA	Electrodes (ECG), PPG	HRV, PTT	Chest (ECG), wrist (PPG)	Detection of sleep-disordered breathing with sympathetic overdrive	Sympathetic overdrive during sleep reflected in EEG, ECG signals, including HR acceleration, PTT decrease
Murali et al., 2003 [56]	Review	Electrodes (EOG, EEG, EMG, ECG), PPG finger cuff	BP, HRV, EOG, EEG, EMG, ECG, RR	Head (EEG, EOG, EMG), chest (ECG), finger (PPG)	Imaging of autonomic functions during normal and pathological sleep	Sleep, sleep stage, and arousal linked to changes in neural circulatory control, hemodynamics measured by various signals including BP, HR, HRV
Nakayama et al., 2019 [66]	Validation, PhysioNet apnea-ECG database	Electrodes (ECG)	ML algorithm trained on multiple HRV features (meanNN, SDNN, RMSSD, Total Power NN50, pNN50, LF, HF, LF/HF, LFnu, HFnu)	Chest	Classification of OSA vs non-OSA	HRV features in ML algorithm detect OSA with 76% sensitivity and 92% specificity, imposing CV risk assessment
Ozegowski et al., 2007 [58]	Observational cross-sectional, n = 74 suspected of sleep-related breathing disorders	Electrodes (ECG)	ML algorithm trained on EDR features (mean EDR amplitude, STD of EDR amplitude, PSD of EDR signal) and HRV features	Chest	Screening of sleep-disordered breathing by prediction of AHI-index based on ECG morphology and HRV in home environment	Early detection of sleep-related breathing disorders by monitoring autonomic responses might improve the prognosis in patients with CV disorders
Park and Choi, 2019 [57]	Review	Electrodes (ECG, EEG, EMG, EDA), PPG, BCG, PAT, accelerometer, radar	HR, BP, RR, PAT, HRV, actigraphy	Chest (ECG), wrist (PPG), finger (PPG, PAT), bedside (mobile phone, camera), ear (EEG), beneath bed’s legs (load cell); under mattress at dorsal surface (PVDF- or EMFi film sensor)	Remote sleep monitoring based on sleep-stage-dependent autonomic balance modulation	Devices measure sympathetic overdrive related to AHI, aiding cardiovascular health assessment
Penzel et al., 2002 [67]	Observational cross-sectional, n = 21 OSA and arterial hypertension	PAT	PAT amplitude	Finger	Early diagnosis of sleep-related breathing disorders	Sympathetic overdrive during sleep due to OSA, arterial hypertension detected by hemodynamic changes: BP, HR, arterial tone
Rahman and Morshed, 2021 [68]	Validation, n = 507 healthy, n = 303 mild OSA, n = 190 severe OSA	Electrodes (ECG), PPG finger cuff	AdaBoost classifier trained on multiple features	Chest (ECG), finger (PPG)	Classification of OSA severity	HRV and SpO2 features estimate OSA severity, aiding in cardiovascular risk assessment during sleep
Tong, 2022 [69]	Validation, n = 15 healthy, n = 15 OSA	PPG	HRV: FuzzyEn, SDNN, LF/HF	Finger	Classification of abnormal nocturnal ANS related to OSA	OSA patients exhibit lower FuzzyEn values in HRV, indicating sympathetic overdrive during sleep and potential cardiovascular risks
Urbanik et al., 2019 [70]	Observational cross-sectional, n = 71 suspected OSA	Electrodes (ECG)	HRT: TO, normal TO, TS, normal TS, HRT0, HRT1/2, HRT1, HRT2	Chest	Prediction of AHI-index based on HRT	HRT reflects sinus node, baroreceptor reflex variability, affecting ANS balance, sympathetic/parasympathetic activities, pertinent to CV health
Yang et al., 2005 [71]	Observational cross-sectional, n = 65 OSA	Electrodes (ECG)	HRV: SDNN, pNN50, LF, HF, LF/HF, RMSSD	Chest	HRV analysis for risk assessment of sleep apnea severity	Apnea-induced sympathetic activation linked to cardiovascular risk during sleep-disordered breathing
Yilmaz et al., 2023 [72]	Observational cross-sectional, n = 78 male healthy	PPG	PPG pulse waveform features: PD, Rt, ΔT, Sys Amp, and Dias Amp, Rslope, RI, ΔT_norm, SI	Finger	Imaging of nocturnal vascular health	Nocturnal variation in PPG waveform corresponds to changes in HRV and BP, indicating cardiovascular modulation during sleep

The most common method for monitoring autonomic activity is the analysis of heart rate variability (HRV), which can be derived from inter-beat intervals (IBIs) estimated from heart activity. For this purpose, ECG is considered the clinical gold standard modality [73]. HRV metrics are associated with CV clinical profiles and are strong, independent predictors of survival in heart failure. This underscores the clinical relevance and interventional potential of HRV analysis for managing CV conditions [74].

The analysis of HRV in both the time and frequency domain are the most commonly used methods, as demonstrated in the study by Yang et al. (2005) [71]. They computed metrics in both the time domain (SDNN, SDANN, RMSSD, pNN50) and frequency domain (VLF, LF, HF, LFnu, HFnu, LF/HF). Additionally, they adopted Heart Rate Turbulence (HRT), a phenomenon related to baroreflex-mediated HR adjustments that serves as a counter-mechanism to premature ventricular contractions. HRT involves a brief acceleration in HR, followed by a gradual return to the baseline rate, quantified by turbulence onset (TO), reflecting the initial acceleration of HR, and turbulence slope (TS), describing the subsequent deceleration of HR. The study established HRT as a more reliable marker for sympathovagal balance compared to general HRV-based metrics, as evidenced by a significant inverse relationship between nighttime TS and the severity of sleep-disordered breathing, generally associated with an heightened sympathetic overdrive [71]. This observation was substantiated by Urbanik et al. (2019), who used the same features to predict imbalances in ANS activity, particularly characterized by sympathetic overdrive, as seen in obstructive sleep apnea (OSA) [70].

To assess the adaptive capabilities and stability of the ANS function, Cabiddu et al. (2015) utilized non-linear complexity metrics of HRV, including Sample Entropy (SE), Lempel–Ziv Complexity (LZC), and Detrended Fluctuation Analysis (DFA). These metrics were applied to IBIs derived from the ECG signal obtained from the chest and compared in a group of healthy subjects and obese patients, respectively [61]. Their findings indicated that a decrease in these parameters among the obese group, compared to the healthy controls, was linked to increased sympathetic overdrive, with the most pronounced reduction occurring during the NREM sleep phase [61].

Similarly, Costa et al. (2021) employed ECG electrodes on the chest but focused on Heart Rate Fragmentation (HRF) metrics [54]. Unlike standard HRV metrics, HRF was sensitive to frequent changes in HR acceleration, and it was suggested as a potential indicator of neuro-autonomic and electrophysiological anomalies of the cardiac control system. The extracted HRF metrics included: (1) the percentage of inflection points (PIP), reflecting changes in heart acceleration sign, (2) the average length of accelerative/decelerative segments (ALS), (3) the overall percentage of normal-to-normal (NN) intervals in long segments (PNNLS), and (4) the overall percentage of NN intervals in short segments (PNNSS). In their study, Costa and colleagues found that HRF metrics improved prediction of atrial fibrillation (AF) under sympathovagal imbalance as compared to standard HRV parameters, and that these measures reflected autonomic changes in each sleep stage [54]. Mayer et al. (2019) employed a receiving operating characteristic curve to identify changes in heart rate acceleration (HRa), extracted from the ECG [65]. In addition, they determined the pulse transit time (PTT), which represents the time taken for the pulse pressure waveform to travel from the aortic valve to a peripheral site (i.e., the finger) where the PPG sensor was attached. Both HRa and PTT parameters were able to effectively capture sympathetic overdrive associated with cortical arousal induced by breathing alterations during sleep. Although promising, the PTT metric requires at least two sensors attached to the body to calculate the time delay of the pulse wave travel, which is a potential drawback in RPM systems for daily use [65]. Moreover, solely looking at HRV metrics obtained from the ECG, the study of Ozegowski et al. (2007) also analyzed the slow modulation of the ECG amplitude which corresponds to the frequency of the breathing cycle. Herewith they were able to obtain an ECG-derived respiration rate (EDR) [58].

#### Optical Sensors

Signals obtained from optical sensors such as the PPG, and the PAT allow continuous monitoring of pulsatile variations in peripheral blood flow, offering an indirect measure of successive IBIs. Moreover, since the ANS controls blood vessel dilation and constriction, which affects blood vessel tone, these changes can also be an indirect measure of ANS function as well as endothelial function [72,75].

For the PAT sensor there is a unified pressure field incorporated (typically with a cuff around the fingertip). Herewith, the distention of the veins is prevented allowing to assess the arterial tone and volume changes in the peripheral arteries, and therefore is able to assess the arterial stiffness [67]. This approach was explored in the research conducted by Penzel et al. (2002) [67]. Their study revealed that, beyond alterations in HR and BP associated with arterial hypertension, sympathetic overdrive could also be detected through characteristics in PAT amplitude [67]. Furthermore, Yilmaz et al. (2023) extended this physiological rationale to analyze signal changes in the PPG waveform, paralleling variations in HRV and BP, which are indirect measures of arterial elasticity [72]. Similarly to Mayer et al. (2019), the study by Carek and Holz (2018) used the PPG signal in combination with BCG on the femoral artery to measure PTT [62,65]. They utilized PTT to estimate continuous BP by applying the rationale of the Moens–Korteweg equation [76], which links arterial stiffness directly to pulse wave velocity.

The PPG signal enables the extraction of various HRV metrics. However, as noted by Baek and Cho (2019), continuous measurement during daily activities, where frequent movement occurs, can lead to inaccurate HRV readings due to motion artifacts [60]. While PPG is reliable for measuring HR in beats per minute, this represents an average over a minute, making it less affected by movement. In contrast, accurate HRV analysis requires detecting IBIs with at least 1-second precision, making it more sensitive to motion disturbances. To address this challenge, Baek and Cho developed a novel HRV metric using a frequency-tracking algorithm based on oscillation equations, allowing HRV analysis even in noisy PPG signals. This method enables the extraction of metrics reflecting ANS modulation, offering a more detailed analysis than average HR measurements in wearable devices [60].

#### 3.2.2. Non-Intrusive Modalities

In addition to obtaining the metrics reflecting ANS from the above-mentioned wearable modalities, the study by Jung et al. (2016) demonstrated the feasibility of extracting these parameters from non-intrusive modalities. They introduced contact-free BCG sensors integrated into the bed [63]. BCG captures the repetitive motions of the human body resulting from the abrupt ejection of blood into the vessels within each heartbeat. Consequently, it becomes feasible to calculate IBIs, representing the time between these blood pulses, and extract HRV from a non-contact BCG system. The study compared systems incorporating load cells beneath the four bed legs, as well as film sensors positioned beneath the mattress at the dorsal surface of the sleeping subject. Jung et al. (2016) demonstrated that HRV metrics in the time (SDNN, RMSSD, NN50, pNN50), frequency (LF, HF, LF/HF) and nonlinear ( SD1, SD2, SD1/SD2) domains could be extracted successfully [63]. However, challenges arose in signal acquisition due to motion artifacts. Additionally, the study highlighted that in the frequency domain, the LF-power spectrum exhibited the highest predictive power for the oxygen desaturation index (ODI) in a population of subjects with nocturnal and non-nocturnal hypoxemia, suggesting its association with underlying sympathetic overdrive [63].

Matar et al. (2018) reviewed technologies for unobtrusive sleep monitoring, focusing on cardiac, respiratory, and movement activities to extract features like HRV, respiration rate (RR), and posture patterns [55]. They examined the use of infrared and color cameras to capture body posture, detect arousal, and generate remote PPG (rPPG) signals for non-intrusive heart rate measurement. The study also explored the potential of smartwatch designs with integrated PPG and accelerometry for similar data collection. A key trade-off was identified: while cameras offered unrestricted sleep monitoring, the smartwatch’s PPG sensor functioned even when skin was covered, though it provided less detailed posture information. Both approaches struggled with motion artifacts in pulse signals [55].

Radar technology for HR extraction and unobtrusive BCG, discussed by Jung et al. (2016), encountered difficulties in signal processing and motion artifacts [63]. These technologies were applied for sleep staging, based on sleep-stage-dependent variations in autonomic function, assessing sleep quality, and detecting breathing-related issues associated with heightened sympathetic drive. Matar et al. emphasized the appeal of contactless measures while acknowledging limitations in detectable motion range and sensitivity to noise. They also underscored the need to validate novel systems against gold standard clinical devices, such as ECG for HRV estimation, in comparison with signals from emerging technologies like PPG, rPPG, BCG, etc. [63].

Similarly to Matar et al. (2018), Park and Choi (2019) reviewed the latest technological advancements in remote sleep-monitoring. In their review they not only took into account the previously mentioned unobtrusive measures but also considered additional systems, providing a comprehensive overview of the strengths and weaknesses of devices designed to measure sympathetic overdrive during sleep [55,57]. Despite exploring various modalities, the review found that the most accurate results were consistently achieved with IBI-based metrics (e.g., HRV, HRT, HRF) obtained through ECG. This underscores the ongoing necessity to enhance signal acquisition in other modalities that lean towards unobtrusiveness [55,57].

#### 3.2.3. Multiple Modalities and Artificial Intelligence

In addition to acquiring metrics from a single modality, Lee et al. (2020), and Rahman and Morshed (2021) demonstrated the capability of training ML models to identify autonomic dysregulation based on metrics from multiple modalities [64,68]. In particular, Lee et al. (2020) employed a neural network trained on various metrics (mean, min, max, and std) derived from the six-channel EEG positioned on the head, HRV metrics in the frequency domain (LF, HF, and LF/HF) obtained from the ECG on the chest, and mean and min Peripheral Oxygen Saturation (SpO2) values from a pulse-oximeter on the finger [64]. This study showcased the potential of utilizing AI models to integrate multiple peripheral biosignals, providing a more comprehensive understanding of ANS regulation during sleep. The neural networks, trained on these features, successfully classified sleep-disordered breathing with an increased efficiency in respect to using only SpO2 signal and the derived apnea hypopnea index (AHI), demonstrating their ability to finely distinguish between normal ANS regulation and cases with sympathetic overdrive. This finding suggests that the use of an obtrusive EEG cap is no longer essential for classifying sleep disorders. This view was empowered by Rahman and Morshed (2021) who trained a ML model (AdaBoost classifier) on features obtained from the pulse-oximeter sensor, including HRV metrics and SpO2 saturation, and showed their discriminating power to classify OSA severity using an everyday wearable [64,68].

Despite the advantages of ML-based algorithms in interpreting various metrics and modalities, Tong (2022) acknowledged the limited computing power of some smart devices [69]. Consequently, Tong developed a method that circumvents the need for AI networks to classify abnormal autonomic responses. This approach involved applying a Fuzzy Entropy (FuzzyEn) algorithm to the IBIs. Tong validated that a lower FuzzyEn value indicates a lower degree of confusion or higher repetition patterns in HRV signals, reflecting sympathetic overdrive during sleep. As a result, this method could effectively distinguish between OSA and healthy sleep [69].

In addition to exploring various modalities and metrics for monitoring autonomic responses during sleep, the review by Murali et al. (2003) emphasized the impact of sleep, sleep stages, and arousals on neural circulatory control, consequently influencing the measured ANS response both in physiological and pathological sleep [56]. The study employed standard polysomnography (PSG) modalities, including EEG, ECG, EOG, and EMG, along with PPG and blood pressure measurements. It compared signal metrics across different sleep stages and wakefulness, revealing significant variations in the signals during these states. This suggests that accounting for these effects and extracting measures specific to different sleep stages can have significant implications in RPM and its CV implications. Additionally, comparing ANS responses during wakefulness versus sleep, or across different sleep stages, may provide new insights into mapping physiological versus pathological ANS function [56].

### 3.3. Alterations of Nocturnal Autonomic Function and CV Implications

Sleep is a natural, periodic suspension of consciousness during which processes of rest and restoration occur. The cognitive, reparative, and regenerative functions of sleep are essential for maintaining health and homeostasis [56]. Dysfunction in autonomic CV regulation can impact sleep physiology [77]. Moreover, sleep disorders can result in autonomic dysfunctions and impaired CV control, as observed in OSA [78]. Studies reviewed indicated that CV dysfunction and sleep disturbances are frequently associated, highlighting a potential downward spiral. Understanding the mechanisms of this interplay and the underlying autonomic dysregulation may potentially lead to effective intervention strategies.

#### 3.3.1. Sleep-Related Breathing Disorders and Autonomic Dysfunction

As mentioned earlier, sleep disorders can lead to autonomic dysfunctions and impaired CV control, as seen in sleep-related breathing disorders like OSA. This review found broad agreement among the included studies that patients with OSA are at increased risk of CV diseases. The heightened sympathetic drive is considered one of the underlying mechanisms and is linked to various CV pathologies, including coronary artery disease, myocardial ischemia, chronic heart failure, cardiac arrhythmias, diabetes mellitus, and stroke (Jung et al. (2016), Lee at al. (2020), Mayer et al. (2019), Nakayama et al. (2019), Ozegowski et al. (2007), Park and Choi (2018), Penzel et al. (2002), Rahman and Morshed (2021), Tong (2022), Urbanik et al. (2019), and Yang et al. (2005)) [57,58,63,64,65,66,67,68,69,70,71]. However, the pathophysiological mechanisms leading to CV diseases in OSA are complex and not fully understood. It is believed that increased sympathetic nervous system activity due to breathing disruptions during sleep alters cardiac autonomic regulation, contributing to the development of CV diseases.

The studies of Yang et al. (2005) and Urbanik et al. (2019) tried to obtain a better understanding by using HRT, an important prognostic indicator of the autonomic dysfunction in CV diseases reflecting perturbations of arterial BP after ventricular premature contractions [70,71]. Both studies were finding that alterations in nighttime HRT correlate with the severity of sleep-related breathing disorders like OSA [70,71]. The studies of Jung et al. (2016), Nakayama et al. (2019), Ozegowski et al (2007), Rahman and Morshed (2021), and Tong (2022) all used different analysis methods of the HRV, showing that OSA is related to increased repetition patterns between heartbeats during the night indicating a sympathetic overdrive [58,63,66,68,69]. Moreover, using these analyses could allow for classification of the AHI, and therefore serve as an efficient and more accessible approach to detect sleep-related breathing disorders compared to the PSG which requires much time, qualified staff and the necessity of the patient’s hospitalization, which makes it costly; however, herewith potentially allowing for early detection and redirecting the developing course of CV risk. This topic has also been discussed in the review of Park and Choi (2019) where they see that the autonomic modulation during sleep involving the EEG during the PSG could be recorded using several less obtrusive CV measurements including the ECG, PPG, BCG, and PAT making sleep monitoring easier and simpler [57].

#### 3.3.2. Altered Nocturnal Autonomic Modulation Reveals Cardiovascular Risk

As highlighted in the reviews by Murali et al. (2003) and Matar et al. (2018), sleep-related changes in CV function result from a complex interplay between central autonomic influences and CV reflexes [55,56]. Sympathetic control of CV function progressively decreases from wakefulness to deep non-rapid eye movement (NREM) sleep, while parasympathetic tone remains in general dominant throughout most of the sleep period [55,56]. Different studies highlighted how this nocturnal modulation of autonomic activity can be altered in the clinical population. Therefore, targeting specific metrics during different sleep stages could be an effective strategy for identifying early markers of CV disease progression.

Murali et al. (2003) described multiple mechanisms of nocturnal autonomic dysfunctions and CV activity, including the following points [56]: (1) Coronary circulation and sleep: physiological changes during rapid eye movement (REM) sleep may be severely disrupted in individuals with pre-existing coronary artery stenosis, which could explain the association between REM sleep and nocturnal cardiac ischemia; (2) HR control during sleep after myocardial infarction: normally, HR accelerates with inspiration and decelerates with expiration to accommodate increased venous return during lung expansion. This variability, indicative of good cardiac health, causes a decrease in the LF/HF ratio of the RR interval in NREM sleep. However, in post-myocardial infarction patients, the expected NREM-related decrease in the LF/HF ratio is absent and actually increases, followed by a further increase during REM sleep, indicating inappropriate sympathetic dominance and a loss of sleep-related vagal activation; (3) BP and sleep: in healthy normotensive individuals, BP typically declines by 10–20%, a phenomenon known as ‘dipping’. However, emerging evidence shows that the absence of nocturnal BP decline ‘non-dipping’, often seen in obese and OSA patients, or an excessive nocturnal BP decline ‘extreme dipping’, has significant CV implications linked to central ischemia [56]. Cabiddu et al. (2015) demonstrated decreased HRV complexity during NREM sleep, indicating sympathetic overactivity in these patients [33]. Monitoring during NREM sleep is crucial to assess baroreflex function, where baroreflex sensitivity should increase to help maintain a slow HR despite the decrease in BP. Additionally, Costa et al. (2021) demonstrated that monitoring fragmented sinoatrial dynamics using HRF during sleep is associated with the incidence of atrial fibrillation [54]. This emphasizes the importance of sleep-stage-specific ANS monitoring to identify and understand the early markers and mechanisms of CV disease.

#### 3.3.3. Importance of Continuous Monitoring

Monitoring in the home environment can give a more complete picture of heart health, and potentially detect masked dysfunctions like hypertension, which is only evident outside the clinic. As mentioned by Carek and Holz (2018), the most common form of home monitoring of the HR and the BP involves an oscillometry-based BP cuff which the user needs to attach multiple times a day while holding still as the cuff self-inflates [62]. The cuff measures the systolic and the diastolic BP as well as the HR. It is an inexpensive, accurate, and patient-friendly form of RPM. However, this method is limited to tracking of spot measurements during the day, when this obtrusive method of tracking is tolerable. Moreover, it is not possible to map the complete circadian rhythm where in a healthy person the BP tends to dip during the night (10–20% below daytime values), while extreme dipping or non dipping is associated with CV events as mentioned earlier. Herewith highlighting the advances of continuous monitoring in achieving a holistic understanding of the subject, its cardiac autonomic regulation, and e.g., hypertension management.

In the studies by Carek and Holz (2018) and Yilmaz et al. (2023), an initial technical approach to continuous BP monitoring was made by correlating optical reflections from the pulse wave using an optical sensor, such as the PPG [62,72]. This method measures the pulse wave as it propagates across the body, utilizing the PTT. Simultaneously, they were collecting HR and HRV metrics to obtain a broader understanding of the continuous cardiac autonomic regulation. The study of Penzel et al. (2002) did not apply the PTT, but integrated a continuous pressure field around the finger and used the PAT signal to correlate this to the BP [67]. Additionally they used the optical sensor to detect changes in HR to detect hemodynamic changes related to arterial hypertension. However, as highlighted in the study of Baek and Cho (2019), the optical sensors still have a technical challenge where they need to cope with motion artifacts [60]. However, they propose an algorithm more robust to obtain a more accurate pulse wave signal. Therefore, current developments in the field are promising towards continuous cardiac autonomic monitoring.

### 3.4. Feasibility Assessed by Metric Compatibility, Obtrusiveness, Data Accuracy, Continuity, and Practical Considerations

This review assessed various modalities and metrics for RPM of nocturnal ANS activity. Table 2 compares different modalities based on metric compatibility, obtrusiveness, data accuracy, continuity, and practical aspects crucial for long-term monitoring, such as patient comfort, compliance, and economic costs.

#### 3.4.1. Electrodes

Electrodes, particularly ECG systems, provide the most accurate and continuous data for HRV evaluation and other measures. However, their direct skin contact and requirement for precise positioning make them the most obtrusive option, often causing discomfort or skin irritation during long-term use. While high data accuracy is beneficial, the invasiveness reduces patient compliance over time unless integrated into more wearable forms like bands or earpieces. The trade-off is that dry electrodes have lower conductance, resulting in poorer data quality [79]. Recent advancements in wearable electrode technology are focused on improving materials and AI integration for better performance [80]. Economically, electrodes can be cost-effective when used in clinical settings, but repeated usage in home environments might incur ongoing costs for consumables (e.g., adhesive patches).

#### 3.4.2. PPG

PPG sensors, commonly worn on the wrist or finger, offer a balance between accuracy and comfort. They provide HRV, SpO2, and pulse waveform measures with moderate accuracy, especially when movement artifacts are minimized [81]. Liu et al. (2021) demonstrated that PPG-derived features can distinguish between various ANS activation patterns with an 80% classification accuracy [75]. The non-intrusive nature of PPG makes it suitable for long-term monitoring, though wrist placement can cause motion artifacts. Economic costs are relatively low since PPG is widely integrated into consumer devices like smartwatches, but maintaining accuracy may require advanced algorithms [82].

#### 3.4.3. PAT

PAT sensors measure arterial pulsatile volume changes with good accuracy for HRV-related metrics, but they require a cuff that applies uniform pressure, which can be uncomfortable for long-term use. Penzel et al. (2002) highlighted that while the PAT signal correlates with BP, it cannot replace invasive BP measurements [67]. The cuff’s discomfort makes PAT less suitable for continuous RPM, particularly during sleep, and its cost is higher due to the specialized equipment required [67].

#### 3.4.4. Unobtrusive BCG

BCG sensors are embedded into objects like beds or chairs, providing an unobtrusive option for long-term monitoring. They measure HR, RR, and body movements with moderate accuracy but face challenges in precise HRV measurement due to motion artifacts [83]. BCG sensors are suitable for continuous nighttime monitoring, but data is typically collected in intervals. These sensors are integrated into furniture, resulting in high upfront costs, but they ensure high patient compliance due to their non-intrusive nature [55,57,62,63].

#### 3.4.5. Cameras

Both RGB and IR cameras offer contactless monitoring, enhancing patient comfort and compliance. RGB cameras can provide accurate HR measures under good lighting conditions and limited subject movements [84]. IR cameras are less accurate due to single channel processing [85,86]. Both camera types are suitable for interval monitoring during sleep but are limited by motion artifacts. The cost of camera-based systems varies depending on the technology, but they offer a feasible solution for patients requiring contactless monitoring [26].

#### 3.4.6. Radar

Radar technology enables non-contact measurements of HR and RR, even when the subject is covered by a blanket, unlike cameras that require skin exposure. This makes radar a promising option for unobtrusive monitoring. However, its accuracy can be influenced by environmental factors and motion artifacts [87,88]. Radar is particularly suitable for nighttime monitoring with limited movement, but it tends to be more expensive.

#### 3.4.7. Accelerometer

Accelerometers, typically integrated into wearable devices, provide accurate data on body movements and sleep patterns. Park and Choi (2019) note that actigraphy, which uses accelerometer data, is widely accepted for sleep assessment but has limitations in detecting wakefulness due to motion inactivity [57]. While effective for monitoring nighttime arousals through body movements, these devices do not measure direct autonomic responses. However, they are economically feasible and highly compliant due to their integration into consumer-grade devices like smartwatches.

### 3.5. Comparison of Metrics for Autonomic Dysregulation Detection and Cardiovascular Monitoring

When monitoring CV health and detecting autonomic dysregulation, each metric (HR, HRV, pulse waveform (PPG/PAT), BP, SpO2, RR, body movements, and EEG-derived neural activity) offers distinct advantages and challenges. These metrics differ in terms of accuracy, reliability, and feasibility for continuous, non-invasive monitoring, especially when used to detect subtle autonomic changes linked to sleep-related CV dysfunctions such as OSA, hypertension, or heart failure. Below is a comparative analysis of each metric’s role in this context.

#### 3.5.1. Heart Rate

Accuracy: HR, when measured using ECG, is highly accurate, providing reliable time-varying information about cardiac function. PPG-based HR measurements are also accurate but more sensitive to motion artifacts [89]. Reliability: As a fundamental metric, HR has high clinical reliability for assessing CV health and autonomic dysregulations [89]. Autonomic Relevance: While HR changes reflect autonomic shifts (e.g., increased sympathetic activity), it provides limited insight into parasympathetic tone without HRV analysis.

#### 3.5.2. Heart Rate Variability

Accuracy: HRV is the gold standard for assessing ANS function in a non-invasive way, particularly when derived from ECG. PPG-derived HRV has moderate accuracy but can suffer from decreased precision due to motion artifacts or poor signal quality [90]. Reliability: HRV reliably reflects shifts between sympathetic and parasympathetic activity, making it essential for monitoring autonomic dysregulation during sleep, stress, or recovery phases [90]. Autonomic Relevance: HRV is directly linked to ANS modulation. Reduced HRV is a strong indicator of sympathetic overdrive, especially in sleep disorders like OSA.

#### 3.5.3. Pulse Waveform (PPG and PAT)

Accuracy: PPG accurately measures HR and SpO2 but struggles with motion artifacts. PAT, which measures arterial stiffness and serves as a proxy for BP, has moderate accuracy in detecting peripheral vascular changes linked to autonomic regulation [91]. Reliability: PPG and PAT are reliable for continuous HR and BP estimates. However, artifacts from movement and ambient light can reduce consistency [91]. Autonomic Relevance: Pulse waveforms, both PPG and PAT, reflect vascular tone and BP regulation, making them useful in identifying autonomic dysfunctions such as non-dipping BP patterns or sympathetic activation.

#### 3.5.4. Blood Pressure

Accuracy: Cuff-based BP measurements offers highly reliable readings of systolic and diastolic BP. Cuff-less methods (e.g., PAT) provide moderate accuracy but are improving with advances in algorithms [92]. Reliability: BP is a cornerstone in CV monitoring. The absence of a normal nocturnal BP dip or the presence of extreme dipping patterns is a strong marker of autonomic dysregulation, particularly in hypertensive or OSA patients [92]. Autonomic Relevance: BP regulation, particularly dipping patterns during sleep, is directly influenced by autonomic function, specifically sympathetic activity at night.

#### 3.5.5. SpO2

Accuracy: SpO2 is generally accurate when measured using PPG, though it can be affected by motion artifacts or poor sensor placement [93]. Reliability: SpO2 is highly reliable for detecting oxygen saturation, which is critical for diagnosing and monitoring OSA and other sleep-related breathing disorders [93]. Autonomic Relevance: SpO2 levels reflect respiratory and cardiovascular coupling. Drops in SpO2 during sleep (desaturation events) often signal autonomic dysregulation.

#### 3.5.6. Respiration Rate

Accuracy: RR can be accurately measured with PPG, PAT, or accelerometers, but indirect methods may offer lower precision [94]. Reliability: Continuous monitoring of RR is reliable, though body movement can reduce accuracy [94]. Autonomic Relevance: RR is closely linked to autonomic function, with abnormalities potentially indicating autonomic dysregulation, respiratory, or cardiovascular issues.

#### 3.5.7. Neural Activity (EEG)

Accuracy: EEG offers around 85% sensitivity in detecting autonomic dysfunctions [95]. Reliability: EEG is reliable for short-term monitoring, but long-term use can be affected by artifacts. It is less commonly employed for continuous autonomic monitoring [95]. Autonomic Relevance: EEG detects autonomic dysregulation by tracking changes in brain activity patterns, relevant to conditions like epilepsy and sleep disorders.

#### 3.5.8. Body Movements

Accuracy: Accelerometers and gyroscopes achieve around 95% accuracy in detecting body movements [96]. Reliability: Continuous monitoring of body movements is highly reliable, although accuracy depends on sensor placement and calibration [96]. Autonomic Relevance: Body movements provide indirect insights into autonomic function, particularly in detecting sleep disorders and physical activity levels.

#### 3.5.9. Summary

In autonomic function monitoring with a focus on CV implications, HRV (via ECG) and BP emerge as the most reliable metrics, offering both high accuracy and direct relevance to autonomic function. While EEG provides valuable insights into sleep-stage-specific autonomic shifts, it is less practical for continuous home monitoring. PPG and PAT sensors are more user-friendly for continuous home use, though their accuracy can be affected by motion artifacts. A combined multimodal approach that integrates HRV (measured via ECG or PPG), BP, and SpO2 monitoring offers a comprehensive solution for assessing autonomic regulation of the heart, vascular tone, and respiratory function. This integration is especially valuable for detecting autonomic dysregulation and CV risks. A visualization of each metric, evaluating its accuracy in measuring the target physiological variable, reliability for continuous monitoring, and relevance to ANS function is provided in Table 3.

### 3.6. Risk of Bias Assessment and Quality Appraisal

**Figure 3:**
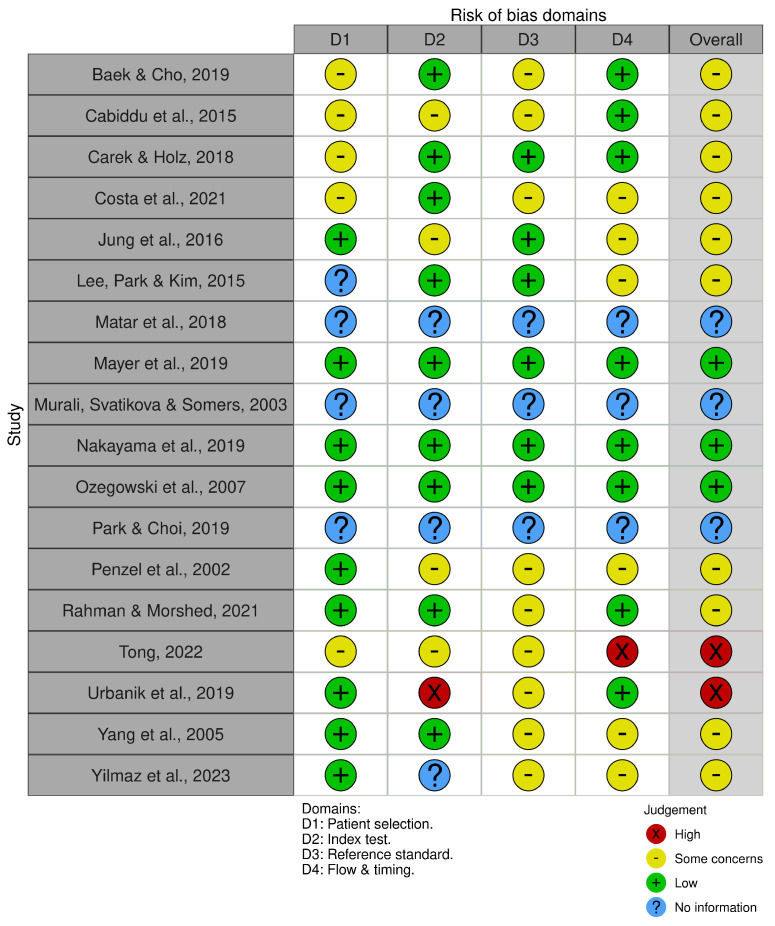
Evaluating trial quality and reporting on the studies by Baek and Choi (2019) [60], Cabiddu et al. (2015) [61], Carek and Holz (2018) [62], Costa et al. (2021) [54], Jung et al. (2016) [63], Lee et al. (2020) [64], Matar et al. (2018) [55], Mayer et al. (2019) [65], Murali at al. (2003) [56], Nakayama et al. (2019) [66], Ozegowski et al. (2007) [58], Park and Choi (2019) [57], Penzel et al. (2002) [67], Rahman and Morshed (2021) [68], Tong (2022) [69], Urbanik et al. (2019) [70], Yang et al. (2005), and Yilmaz et al. (2023) [72]. Risk of bias assessment using the QUADAS-2 tool [53] for primary diagnostic accuracy studies within systematic reviews across four domains.

The summary of the risk of bias, as illustrated in Figure 3, adheres to the revised Cochrane risk of bias tool for primary diagnostic accuracy studies within systematic reviews (QUADAS-2) [53]. It is important to note that, in the case of the included review studies by Matar et al. (2018) [55], Murali et al. (2003) [56], and Park and Choi (2019) [57], the option ’No information’ was applied across all four domains. This decision was made because these studies do not qualify as primary diagnostic accuracy studies.

Notably, two studies [69,70] demonstrated a high risk of bias for the index test and the flow and timing, respectively. Conversely, three studies [58,65,66] revealed a low risk of bias across all four domains, signaling reliable research. For the remaining studies, there were only some concerns and low risks distributed across the domains, presenting no alarming risks overall, as depicted in Figure 3.

**Figure 4:**
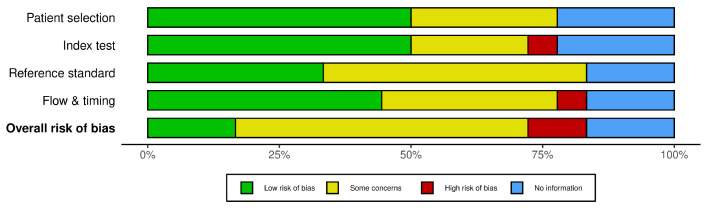
Overview of risk of bias assessment using QUADAS-2 tool [53] across four domains in the included primary diagnostic accuracy studies [54,55,56,57,61,63,64,65,66,67,68,69,70,71,72].

The overall risk of bias, illustrated in Figure 4, predominantly indicates some concerns, primarily attributed to the high percentage of concerns in D3 ’Reference standard’. This might be due to the absence of a standardized reference for sympathovagal balance measurements in RPM systems or technologies compatible with RPM modalities. This suggests a moderate variation in the quality of conduct and reporting.

## 4. Discussion

This systematic review’s objective was threefold: (i) screening the literature to categorize and describe current modalities and metrics for nocturnal ANS activity monitoring; (ii) emphasizing potential CV implications associated with alterations in the nocturnal autonomic function; (iii) assessing the feasibility of the identified modalities for daily life applications in respect to metric compatibility, obtrusiveness, data accuracy, continuity, and practical considerations. As such, literature was examined to review all the potential modalities and metrics capable of continuously assessing nocturnal autonomic function, trying to provide an integrated framework encompassing their integrability in RPM systems as well as their potential implications in CV risk assessment. Herewith distinguishing itself from the other reviews included in this study that were focusing on advancements in unobtrusive sleep monitoring, comparing traditional PSG methods with novel approaches as was performed by Matar et al. (2018) and Park and Choi (2019) on the sole understanding of the mechanisms linking nocturnal autonomic function and CV physiology and pathology, but disregarding the necessity of remote monitoring technologies as was examined by Murali et al. (2003) [55,56,57].

The key findings of this study can be summarized as follows: (1) Various modalities for monitoring nocturnal ANS activity were identified, including electrodes (EEG, ECG, PSG), optical sensors (PPG and PAT), non-intrusive BCG, cameras (RGB and IR), radars, and accelerometers. (2) Among different metrics compatible with the observed modalities, those based on HRV as well as those influenced by arterial tone, such as BP, are the most frequently and successfully used for characterizing nocturnal autonomic and vascular dysregulation. (3) Sleep disorders like OSA lead to autonomic dysfunctions and impaired CV control. While on the other hand nocturnal ANS dysfunctions influence sleep physiology and CV control, exacerbating each other in a downward spiral. Moreover, alterations in the physiological modulation of autonomic activity during the night represent a further prodromal sign of serious CV implications. This evidence clearly highlights the need of continuous ANS mapping, with potential sleep-stage-specific monitoring. (4) Electrodes are deemed the most accurate for continuous HRV monitoring but are also the most intrusive. Optical sensors show promise for multimodal applications, including HRV, SpO2, arterial stiffness, PTT, and correlations to BP. (5) Unobtrusive measures such as BCG, cameras, and radars are capable of accurately estimating HR and RR, but continuous HRV monitoring remains a challenge with these methods.

### 4.1. Challenges and Opportunities in Standardizing Metrics for Remote Autonomic Dysfunction Assessment During Sleep

Continuously mapping autonomic activity and identifying the pathways linking it to CV control represents a fundamental process for gaining a better insight in the mechanisms underlying the development of sustained sympathovagal imbalance and their potential relationship with sleep disorders. Both conditions worsen the prognosis and progression of CV diseases, underscoring the importance of identifying prodromal states of sympathovagal imbalance for early diagnosis and timely intervention. However, the optimal metrics for distinguishing between physiological and pathological autonomical states remains unclear across different applications. This is compounded by the lack of standardized methods for assessing nocturnal autonomic function, making it difficult to interpret results across studies. This challenge is highlighted by the risk of bias in many studies in the ’Reference standard’ domain. The lack of a universally accepted reference standard poses a significant challenge for meaningful comparisons between metrics, particularly since no established reference standard currently exists for remotely monitoring sympathetic overdrive in a home setting. An avenue for testing the discriminative capacity of these metrics in assessing sympathovagal imbalance could involve validation against direct measurements of sympathetic nerve activity through microneurography [97]. This technique, involving the insertion of a fine electrode into peripheral sympathetic fibers, offers unparalleled accuracy but is invasive, time consuming, and detrimental for sleep quality, limiting its use in many studies [97].

Among the most frequently reported metrics for characterizing autonomic function in our review, HRV and BP were highlighted. Carter’s review (2019) exploring the impact of existing microneurography studies over 50 years, reported some significant correlations between the HRV and BP metrics and more invasive microneurography [98]: reduced BP correlated significantly with reduced muscle sympathetic nerve activity [99], and enhanced coupling of muscle sympathetic nerve activity and HRV and BP variability during head-up tilt compared to rest [100]. Moreover, they highlighted that for HRV, the laboratory of Dwain Eckberg critically appraised sympathovagal balance in response to the study of [101], who coupled spectral analysis of HRV directly to sympathetic activity obtained from microneurography. Eckberg (1997) emphasized that while HRV can be used to understand autonomic activity and for CV risk stratification, caution is needed when using spectral analysis of HRV to quantify a balance between sympathetic and vagal neural outflows [7]. All these results refer to autonomic activity during wakefulness. Regarding nocturnal BP, studies by Somers et al. (1995) and Murali et al. (2003) documented increased muscular sympathetic nerve activity and BP in patients with OSA during the night [43,56]. These findings confirm the effectiveness of continuous BP measurements for monitoring autonomic dysregulation. Additionally, they demonstrated that continuous positive airway pressure therapy reduces nocturnal muscular sympathetic nerve activity and BP in OSA patients. This indicates that airway obstruction causes sympathetic overdrive, and its alleviation reduces this overdrive. Continuous BP measurements effectively reflect these sympathetic responses, making BP monitoring a critical target for continuous CV risk assessment and for evaluating intervention strategies. Hence, findings must be interpreted cautiously, necessitating comparison with microneurography measurements to evaluate the validity and reliability of metrics describing sympathovagal imbalance. This highlights the critical importance of validating metrics alongside autonomic dysregulation and control, selecting the most appropriate ones, and thereby laying the groundwork for their integration into an RPM system.

### 4.2. Modalities for Autonomic Function Evaluation with Potential for RPM

To identify the most promising modalities for defining an RPM system for long-term home monitoring of a subject’s autonomic state, a trade-off between obtrusiveness and data accuracy and continuity must be made. This involves considering the specific metrics adopted and their implications for CV risk assessment. As previously mentioned, HRV and BP are the most commonly reported metrics for autonomic monitoring with potential integration into daily-use RPM systems. BP measurements have shown the capacity to directly reflect nerve sympathetic changes. However, the development of non-invasive, cuffless, and continuous BP estimations is a promising yet challenging field. It involves the acquisition of multiple signals, multiple sensor placement on the body, and individual factors, all of which can affect BP estimation accuracy. Conversely, technologies for continuous HRV measurements, along with advancements in signal processing and AI integration, offer promising solutions for accessing continuous HRV data in daily settings. These technologies range from minimally obtrusive devices like bands and watches to non-obtrusive options such as BCG, cameras, and radar.

### 4.3. Heart Rate Variability in RPM

In our review, we identified several modalities capable of evaluating HRV data: electrodes, optical sensors, cameras, radars, and BCG. Electrodes offer the highest data accuracy and are commonly used in clinical settings. However, their obtrusiveness, requiring placement on specific body parts and potentially causing skin irritation, makes them less feasible for daily RPM use.

Many HR-related metrics can be extracted from less obtrusive methods like PPG sensors and even non-intrusive sensors such as cameras, radar, and BCG. The pulsatile signals from these modalities yield HR and HRV characteristics comparable to those from ECG [102,103]. Despite the advantages of PPG sensors, such as ease of use and low cost, their need for direct skin contact limits their feasibility in certain scenarios. This limitation has prompted exploration into non-contact HR and HRV measurement solutions to address mobility constraints and sensor obtrusiveness issues. However, non-contact methods involve a trade-off between data accuracy and obtrusiveness. These methods can experience data loss when subjects move out of sensor range and require advanced signal processing to mitigate motion artifacts [104]. Moreover, motion can lead to the loss of an IBI, distorting the comprehensive understanding of HRV responses. Despite this, the accuracy of these non-intrusive methods is adequate for averaged measures such as HR and RR.

Therefore, we suggest that in scenarios where skin sensor placement is feasible, transitioning to dry electrodes or a PPG sensor integrated into a band-like wearable is currently the most practical approach for HRV-based metric assessment in everyday settings. Portable ECG devices such as AliveCor or MyDiagnostick for smartphones, or smartwatches like the Apple Watch Series 4® with integrated ECG capabilities, provide solutions for recording ECG, effectively balancing data accuracy with sensor convenience [105,106,107,108,109,110,111]. The integration of ECG into smartwatch-like devices enhances accessibility and usability for RPM applications. However, it is important to note that none of the above mentioned devices allow for continuous heart rhythm measurements; instead, users must manually start an ECG recording when they experience symptoms or receive an irregular rhythm notification based on data collected with the PPG sensor [112]. Considering the PPG, despite its limitation of providing only indirect indications of underlying cardiac rhythms, its major advantage relies in the broad integration of this modality into the current day’s smartwatches, allowing a big data stream of continuous HR derived measures and thus the possibility of reconstructing subject’s IBIs, when lost due to motion artifacts using AI [113,114,115].

### 4.4. Continuous Blood Pressure in RPM

In our review, we identified the potential of optical sensors for indirectly assessing BP by analyzing the pulse waveform and its propagation. Carek and Holz (2018) used the PTT from BCG to PPG sensors placed on the legs [62]. Similarly, Park and Choi (2019) discussed the potential of using PTT from an ECG sensor to a PPG sensor at the wrist, as tested by Mayer et al. (2018) [55,57]. Alternatively, pulse wave propagation features can be directly associated with changes in the PPG signal, such as amplitude drop or signal derivative, as demonstrated by Yilmaz et al. (2023) [72]. Penzel et al. (2002) used PAT to correlate pulsatile volume change with BP [67]. The advantage of PAT is that it involves a continuous pressure field around the finger, reducing the signal-to-noise (SNR) ratio and providing a clearer understanding of wave propagation.

### 4.5. Advantages and Disadvantages in Using PPG for Remote Autonomic Function Monitoring

PPG is able to monitor both HRV and BP continuously in a less obtrusive way as the ECG, and the intra-arterial or inflatable cuff, respectively. A significant amount of further information can be extracted from the sole PPG and its derivative waveforms. The systolic peak can be used for HR, the dicrotic notch and the areas before and after can be associated with stroke volume, and slope transit time can indicate hypertension [116,117]. The first derivatives are linked to blood velocity, which in turn relates to BP [116,117]. Moreover, PPG slowing varying features have been associated with variation in autonomic function. In particular, sudden drops in the amplitude of the PPG pulsatile waveform have been suggested to reflect sympathetic activations of the skin vasculature underlying peripheral vasoconstriction [35]. Mean number of drops per hour during a night of sleep have been demonstrated to be a good predictor of CV risk in OSA [118]. Overall, this evidence makes this modality a potential candidate for RPM due to its capacity to extract multiple features able to reflect autonomic function.

Nonetheless, to ensure the reliability of PPG features, the waveform must be of high quality and with a high SNR ratio. To improve the accuracy of continuous monitoring, a comprehensive understanding of the sources of inaccuracies is required, as highlighted by Fine et al. (2021) [119]. They identified several key noise sources and corresponding solutions. Individual variations, such as skin tone, can lead to decreased signal intensity, which can be addressed by selecting the appropriate PPG wavelength. Obesity also results in decreased signal intensity, though currently, there is no solution for this issue. Age can alter the PPG waveform and signal intensity, and this can be resolved through calibration, as can the changes in signal intensity associated with gender. Physiological factors also play a significant role in modifying the PPG waveform. For instance, RR can alter the waveform, but this can be corrected using a high-pass filter. Venous pulsations also affect the PPG waveform, which can be mitigated with high-pass filtering and applying a pressure field. Local body temperature could change signal intensity and can be managed through calibration. Additionally, the body site in which the PPG sensor is applied affects signal intensity and waveform, which can also be calibrated. External factors such as motion artifacts and ambient light can degrade the SNR. These issues can be resolved using filters and secondary sensors. Moreover, optical shielding and applying optimal pressure are essential for maintaining a high SNR without affecting waveform features [119].

### 4.6. Potential Applications of a RPM System Capable to Monitor Nocturnal Autonomic Dysregulation

Our findings highlight the potential of RPM systems capable of assessing nocturnal autonomic dysfunction to facilitate early diagnosis and intervention for a wide range of conditions characterized by sympathetic overdrive, such as systemic hypertension, myocardial infarction, congestive heart failure, stroke and many others [45,46]. Nonetheless, this review clearly showed that nocturnal autonomic assessment results predominantly limited to sleep-related breathing disorders. We emphasize the importance of focusing on nocturnal autonomic activity, leveraging the absence of many confounders (e.g., daily living and work activities) and the ANS dominance over the central nervous system during sleep. This approach could be applied to a broader range of cardiometabolic pathologies related to autonomic dysregulation [14,15,16,17,18,19,20,21,22,23,24,25,26].

In this review, the benefits of monitoring autonomic function during sleep were discussed for various pathologies. For instance, physiological changes during REM sleep may be severely disrupted in individuals with pre-existing coronary artery stenosis. In myocardial infarction, the HRV parameter LF/HF increased during NREM sleep and further increased during REM sleep compared to a decrease in this parameter during NREM for healthy subjects. For systemic hypertension, continuous BP monitoring can help identify extreme dipping or non-dipping profiles. For atrial fibrillation, monitoring the incidence of fragmented sinoatrial dynamics using HRV during sleep was suggested. However, the advantages of sleep monitoring in congestive heart failure were not discussed, despite its potential benefits.

In fact, sleep apnea is a common and serious comorbidity in congestive heart failure, often underdiagnosed [120]. It results in additional sympathetic overdrive during sleep, compounding the persistent sympathetic drive during wakefulness. Implementing a RPM system would allow for a better understanding of the mechanisms linking sleep breathing disorders to autonomic dysregulation in congestive heart failure. This presents a compelling use case, as congestive heart failure is a significant healthcare challenge affecting millions worldwide. Its prevalence is increasing due to improved post-diagnosis survival rates and an aging population [121,122].

For congestive heart failure, RPM technologies capable of capturing ANS dysregulation could offer advantages over traditional non-invasive parameters, as ANS-related parameters change earlier in the cascade of events leading to decompensated congestive heart failure than signs and symptoms [123]. Early detection and intervention, facilitated by monitoring these ANS-related parameters, could potentially mitigate hospitalization and mortality rates. To address these gaps, we propose applying and validating all identified metrics within a single application characterized by sympathetic overdrive, such as congestive heart failure, to assess their ability to distinguish between different ANS states. This approach could provide valuable insights into early detection and intervention strategies for managing congestive heart failure more effectively. Additionally, the potential of sleep interventions is highlighted by studies showing that the diagnosis and treatment of central sleep apnea or Cheyne–Stokes respiration in congestive heart failure patients with continuous positive airway pressure or adaptive servo-ventilation during the night are associated with improved clinical outcomes [124,125,126]. This improvement is believed to be caused by the relief of continuous sympathetic overactivity, restoring the recovering function of sleep through homeostasis.

Another important aspect is the challenge RPM systems face in ensuring data privacy and security. The continuous collection and transmission of sensitive health data introduce potential vulnerabilities, such as data breaches and unauthorized access. Robust encryption protocols, secure data storage systems, and stringent access controls are essential to safeguard patient information. Additionally, ensuring device security and maintaining the integrity of interconnected networks are critical to prevent tampering and unauthorized access. Addressing these challenges is crucial for building trust between patients and healthcare providers, thereby facilitating the effective implementation of RPM systems [127]. Note that the challenges of data privacy and security are related to the company brand and management, not to the metrics and modalities reviewed in this paper.

#### Future Research Directions

Our review underscores a critical gap in current RPM systems and telehealth applications, which predominantly focus on monitoring wakefulness. This focus highlights a significant limitation: the need for systems that offer insights into nocturnal autonomic and circadian regulation. During sleep, the ANS plays a pivotal role that is often overshadowed by the CNS (Central Nervous System) during wakefulness.

The existing monitoring systems primarily cater to sleep staging, sleep quality assessment, and detection of breathing-related sleep disorders. While these systems are valuable, they do not fully capture the intricate dynamics of nocturnal autonomic regulation. Given the ANS’s crucial role in regulating cardiovascular health and maintaining homeostasis during sleep, there is an urgent need for non-intrusive monitoring systems that can assess ANS activity throughout the night without disrupting sleep.

Moreover, the link between sleep disorders, particularly breathing-related disorders, and cardiovascular diseases remains underexplored. Research has not sufficiently examined the nocturnal autonomic changes associated with conditions such as heart failure. This knowledge gap hampers our understanding of the comorbidity between sleep-related breathing disorders (e.g., central sleep apnea) and heart failure. It also limits our ability to develop effective interventions to address the continuous sympathetic overdrive that persists during sleep, a period crucial for restorative processes.

To address these gaps, we propose a road map for advancing nocturnal autonomic regulation research:

- Development of Advanced Monitoring Technologies: Prioritize the creation of non-intrusive technologies that can accurately measure nocturnal ANS activity. Such advancements are fundamental for obtaining reliable data on autonomic regulation and cardiovascular health.
- Longitudinal Studies on Nocturnal Autonomic Changes: Utilize these advanced monitoring technologies to conduct studies that investigate how nocturnal autonomic changes correlate with cardiovascular pathologies. These studies could help identify potential biomarkers for early intervention.
- Integration of Multimodal Data: Investigate the integration of data from various metrics, including HRV, BP, and SpO2, while considering the sleep phases during which these measurements are taken. This approach could offer a comprehensive understanding of autonomic regulation and its impact on CV health.
- Evaluation of Intervention Strategies: Assess and validate therapeutic approaches aimed at decreasing sympathetic activity and improving patient outcomes.
- Validation of Clinical Utility: Confirm the effectiveness and practicality of these monitoring technologies in real-world clinical settings.

## 5. Conclusions

In conclusion, we categorized and described the current methods for nocturnal ANS monitoring identified in the literature. Various modalities were reviewed, including electrodes (EEG, ECG, PSG), optical sensors (PPG and PAT), non-intrusive BCG, cameras (RGB and IR), radars, and accelerometers. Among reported metrics compatible with these modalities, HRV and BP emerged as the most commonly and successfully used metrics. HRV is valuable for understanding autonomic activity and CV risk stratification, although caution is needed when using spectral analysis of HRV to quantify the balance between sympathetic and vagal neural outflows. BP, directly related to sympathetic activity, proves effective for monitoring sympathovagal imbalance, highlighting the importance of continuous BP monitoring for CV risk assessment and intervention evaluation. Secondly, we investigated the connection between nocturnal autonomic dysregulation and CV risk. HRV and BP were noted as critical indicators due to their relationship with both autonomic function and CV health. Studies reviewed indicated that CV dysfunction and sleep disturbances are frequently associated, highlighting a potential downward spiral of health. Understanding the mechanisms of this interplay and the underlying autonomic dysregulation may potentially lead to effective intervention strategies. Given that sleep is a period for homeostasis and recovery, where the baroreflex is reset, targeting specific metrics during different sleep stages could be an effective strategy for identifying early markers of CV risk.

Finally, we assessed the feasibility of the identified methods based on their compatibility with selected metrics, obtrusiveness, data accuracy, continuity, and practical considerations. HRV monitoring appears most feasible due to advancements in dry ECG electrode development for wearables such as bands and watches, which offer minimal invasiveness. PPG, with calibration options and signal reconstruction capabilities, can provide accurate IBIs for HRV analysis. Non-obtrusive methods like BCG and camera systems can be integrated near the bedside, enabling continuous monitoring without the discomfort of wearing devices, though with a trade-off in HRV accuracy. Continuous BP monitoring using non-invasive PPG remains promising yet challenging, with two main research directions: waveform morphology theory and waveform propagation theory. The former requires extensive data and individualized calibration, while the latter involves multiple sensors and faces challenges related to signal acquisition and accuracy.

Overall, while significant advancements have been made in nocturnal ANS monitoring, further research and development are needed to enhance the accuracy, feasibility, and integration of these methods into RPM systems for effective long-term monitoring and management of autonomic function and cardiovascular risk.

## 6. Limitations

We acknowledge that our initial PICO search strategy, which centered on CV implications and RPM during nighttime, may have excluded certain remote monitoring technologies. This focus was intentional, as CV diseases pose a significant global healthcare challenge, affecting millions with increasing prevalence due to improved post-diagnosis survival rates and an aging population [121,122]. We also deliberately prioritized nocturnal monitoring, potentially overlooking daytime technologies like inflatable BP cuffs that are appropriate during the day but too invasive for nighttime use. Nighttime monitoring was chosen with the expectation of obtaining more reliable baseline measurements, as nighttime conditions reduce disturbances from activities such as body movement or leaving sensor-monitored rooms. Additionally, ANS responses are more prominent during sleep, offering clearer measurements less influenced by the CNS, which dominates during wakefulness.

However, we recognize that this narrower focus may have excluded other patient populations that could benefit from ANS monitoring, such as those with insomnia, periodic leg movement syndrome, cognitive impairment, cerebrovascular disorders, and metabolic conditions like obesity and diabetes [2,12,39,40,44,45,46,47].

To address the possibility of missing out on relevant technologies, we conducted an additional search. This time, we repeated the search string shown in Figure 1 but excluded terms such as “Cardiovascular”, “Cardiac”, and “Heart”. From this, we selected the six most recent (from 2021 onward) and most highly cited studies focused on non-invasive wearables and nearables for nocturnal monitoring. Through this extended review, we identified several innovative technologies. For instance, Heenman and Sang (2023) discussed metrics and modalities similar to those in our review, but highlighted an additional technique based on a microphone to extract respiratory rate and detect movement [128]. Kwon et al. (2021) [129] introduced an advanced patch-like device with an integrated stethoscope, developed by Klum et al. [130], that measures left ventricular ejection time and pre-ejection period, critical markers for heart failure, and extracts respiratory rate through lung sound analysis. The device also incorporates ECG for HRV measurement, providing a comprehensive view of sleep physiology. Other cutting-edge technologies include impedance plethysmography [131], which accurately estimates pulse waves at the wrist compared to ECG, and a wearable ultrasound patch [132] that measures tongue thickness, relevant for OSA events. Beyond CV implications, Li et al. [133] explored the relationship between nocturnal sympathetic nervous system activity and cognitive dysfunction in OSA patients, while van Eekelen et al. [134] demonstrated how sleep deprivation affects HRV and PPG markers, emphasizing the value of continuous ANS monitoring during sleep.

These findings show that the metrics and modalities align closely with those in our study. However, our research takes a unique approach by specifically examining these technologies within the context of nocturnal ANS, CV implications, and RPM, a focus largely missing from previous studies. This distinction underscores the importance of our work, as it addresses a critical gap by framing these technologies within CV health monitoring, offering new insights into their potential applications in the field of sleep.

## Figures and Tables

**Table 2:** Feasibility of different modalities for physiological monitoring of autonomic regulation assessed by compatibility, obtrusiveness, data accuracy, continuity, and practical considerations.

Modality	Compatible Metrics	Obtrusiveness	Data Accuracy	Continuity	Patient Comfort and Compliance	Economic Cost
Electrodes	HRV, skin conductance, neural activity	High (skin contact)	High	Continuous	Low (discomfort, skin irritation)	Moderate (for consumables)
PPG sensor	HRV, SpO2, pulse waveform	Low (wrist or finger contact)	Moderate to high (prone to motion artifacts)	Continuous	High (wearable, non-invasive)	Low (widely available)
PAT	HRV, PAT amplitude	Moderate (cuff pressure)	Moderate to high	Interval	Low (pressure discomfort)	High (specialized equipment)
BCG	HR, RR, body movements	Low (no contact)	Moderate (prone to artifacts)	Interval	High (embedded in environment)	High (infrastructure cost)
RGB camera	HRV, RR, body movements	None (contactless)	Moderate (lighting conditions, artifacts)	Interval	High (contactless, non-invasive)	Moderate to high (equipment)
IR camera	HR, RR, body movements	None (contactless)	Low (limited by single-channel processing)	Interval	High (contactless, non-invasive)	Moderate to high (equipment)
Radar	HR, RR, body movements	None (contactless)	Moderate (affected by motion, interference)	Interval	High (contactless, non-invasive)	High (advanced technology)
Accelerometer	Body movements	Low (wearable)	Moderate	Continuous	High (integrated in wearables)	Low (widely available)

**Table 3:** Comparison of metrics for ANS dysregulation detection and cardiovascular monitoring.

Metric	Accuracy	Reliability	Autonomic Relevance
HR	High (ECG), Moderate (PPG, motion-sensitive)	High (Clinically reliable for CV health)	Moderate (Reflects autonomic shifts, but limited without HRV analysis)
HRV	Gold standard (ECG), moderate (PPG)	High (Reliable for ANS shifts)	High (Strong indicator of autonomic modulation, especially sympathetic overdrive)
Pulse Waveform (PPG/PAT)	Accurate (PPG for HR, SpO2), Moderate (PAT for BP)	Moderate (Affected by motion artifacts)	Moderate (Reflects vascular tone and BP regulation)
BP	High (Cuff-based), Moderate (Cuff-less)	High (Cornerstone for CV monitoring)	High (BP dipping patterns strongly reflect autonomic activity)
SpO2	High (PPG, but motion-sensitive)	High (Critical for respiratory disorders like OSA)	Moderate (Desaturation events linked to autonomic dysregulation)
RR	High (PPG, PAT, or accelerometers)	Moderate (Affected by body movement)	Moderate (Linked to autonomic control of respiratory and CV systems)
Neural Activity (EEG)	Moderate ( 85% sensitivity for autonomic dysfunctions)	Moderate (Reliable but affected by artifacts)	Moderate (Tracks brain-autonomic links in conditions like epilepsy, sleep disorders)
Body Movements	High (Accelerometers/gyroscopes, 95%)	High (Depends on sensor calibration/placement)	Low (Indirect insights into autonomic function through activity levels and sleep)

## Data Availability

The data are contained within the article.

## Supplementary Materials

The following supporting information can be downloaded at: https://www.mdpi.com/article/10.3390/bioengineering11101045/s1, Table S1: Details of articles excluded after title and abstract screening.

## Abbreviations

The following abbreviations are used in this manuscript:

ANS	Autonomic Nervous System
CV	Cardiovascular
BP	Blood Pressure
RPM	Remote Patient Monitoring
PICO	Population, Intervention, Comparator, Outcome
ECG	Electrocardiogram
PPG	photoplethysmography
PAT	Peripheral Arterial Tone
BCG	Balistocardiography
EEG	Electroencephalogram
EDA	Electrodermal Activity
EMG	Electromyography
EOG	Electrooculography
PSG	Polysomnography
HR	Heart Rate
HRV	Heart Rate Variability
IBI	Inter Beat Interval
SDNN	Standard Deviation of the NN Intervals
SDANN	Standard Deviation of the Average NN Intervals
RMSSD	Root Mean Square of the Successive Differences
pNN50	Proportion of NN50 divided by the total number of NN intervals
VLF	Very Low Frequency
LF	Low Frequency
HF	High Frequency
LFnu	Normalized Low Frequency
HFnu	Normalized High Frequency
HRT	Heart Rate Turbulence
TO	Turbulence Onset
TS	Turbulence Slope
OSA	Obstructive Sleep Apnea
SE	Sample Entropy
LZC	Lempel-Zive Complexity
DFA	Detrended Fluctuation Analysis
HRF	Heart Rate Fragmentation
PIP	Percentage of Inflection Points
ALS	Accelerative Segments
PNNLS	Percentage of NN Intervals in Long Segments
PNNSS	Percentage of NN Intervals in Long Segments
AF	Atrial Fibrillation
HRa	Heart Rate acceleration
PTT	Pulse Transit Time
EDR	ECG derived respiration rate
PVDF	Polyvinylidene Fluoride
EMFi	Electro-Magnetic Field imaging
ODI	Oxygen Desaturation Index
RR	Respiration Rate
rPPG	Remote Photoplethysmography
IR	Infrared
RGB	Red, Green, Blue
AI	Artificial Intelligence
ML	Machine Learning
MLP	Multilayer Perception
SpO2	Peripheral Oxygen Saturation
AHI	Apnea-Hypopnea Index
FuzzyEn	Fuzzy Entropy
NREM	Non-Rapid Eye Movement
REM	Rapid Eye Movement
SNR	Signal-to-Noise Ratio
CNS	Central Nervous System
